# Supplementary material for: Chromosome Doubling Enhances Biomass and Carotenoid Content in Lycium chinense
Source: Plants (Basel). 2024 Feb 2;13(3):439. doi: 10.3390/plants13030439 (PMC10857560; doi:10.3390/plants13030439)
Supplement: Supplementary file 1 [file plants-13-00439-s001.zip › plants-2813240-supplementary.pdf]

Supplementary Material

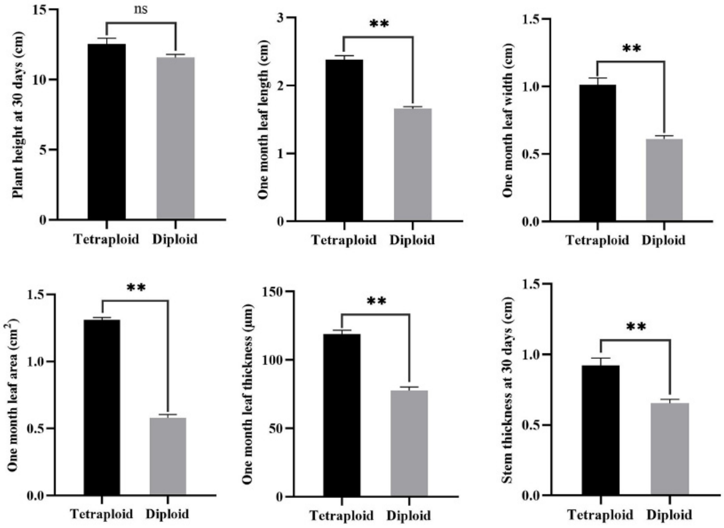

Supplementary Figure 1. Statistical analysis (30 days) of plant height, leaf length, leaf width, leaf area, leaf thickness, and stem thickness of diploids and tetraploids *L. chinense*. **for $P \leq 0.01$, ns for $P > 0.05$.

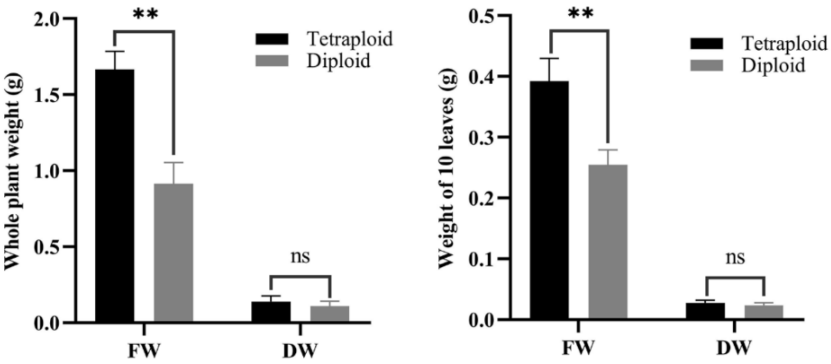

Supplementary Figure 2. Statistical analysis of fresh and dry weights of the whole plants and 10 leaves of diploids and tetraploids *L. chinense*. **for $P \leq 0.01$, ns for $P > 0.05$.

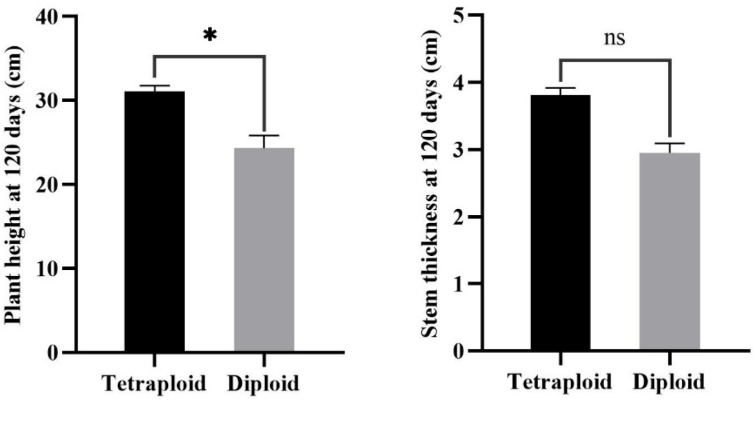

Supplementary Figure 3. Statistical analysis (120 days) of plant height and stem thickness of diploids and tetraploids *L. chinense*. *for 0.01 < *P* ≤ 0.05, ns for *P* >0.05.

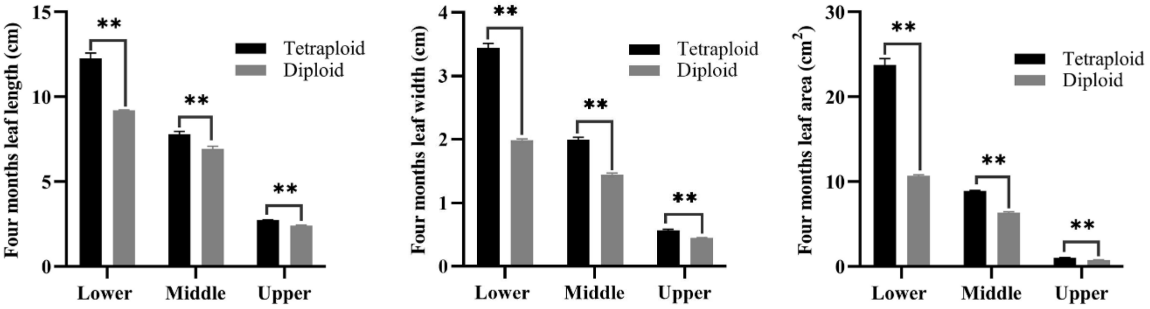

Supplementary Figure 4. Statistical analysis (120 days) of leaf length, leaf width, and leaf area of diploids and tetraploids *L. chinense*. **for *P* ≤ 0.01.
